# Identification and Functional Characterization of Plant MiRNA Under Salt Stress Shed Light on Salinity Resistance Improvement Through MiRNA Manipulation in Crops

**DOI:** 10.3389/fpls.2021.665439

**Published:** 2021-06-17

**Authors:** Tao Xu, Long Zhang, Zhengmei Yang, Yiliang Wei, Tingting Dong

**Affiliations:** ^1^Jiangsu Key Laboratory of Phylogenomics and Comparative Genomics, School of Life Sciences, Jiangsu Normal University, Xuzhou, China; ^2^Department of Applied Biology, College of Agriculture and Life Sciences, Chonnam National University, Gwangju, South Korea

**Keywords:** miRNA, plant, salt stress, tolerance, salinity resistance, crop

## Abstract

Salinity, as a major environmental stressor, limits plant growth, development, and crop yield remarkably. However, plants evolve their own defense systems in response to salt stress. Recently, microRNA (miRNA) has been broadly studied and considered to be an important regulator of the plant salt-stress response at the post-transcription level. In this review, we have summarized the recent research progress on the identification, functional characterization, and regulatory mechanism of miRNA involved in salt stress, have discussed the emerging manipulation of miRNA to improve crop salt resistance, and have provided future direction for plant miRNA study under salt stress, suggesting that the salinity resistance of crops could be improved by the manipulation of microRNA.

## Introduction

Salinity, as a major environmental stress factor, restricts crop growth and yield globally. It is reported that salinity affected a land area as large as 800 million hectares across the globe, accounting for 6% of the land (Abdel Latef et al., 2020; Attia et al., 2021). Approximately 20% of the irrigated soils are affected by salinity stress (Zhao et al., 2013), and 50% of arable land will be affected by 2050 (Butcher et al., 2016). Salt stress leads to changes in metabolic activity, cell wall damage, and cytoplasmic dissolution; it reduces the photosynthetic efficiency, accelerates aging, increases respiratory consumption and toxin accumulation, and eventually results in plant death (Osman et al., 2020; Abdel Latef et al., 2021). It is estimated that salinity can result in $27.3 billion in agricultural damage every year (Qadir et al., 2014). On the other hand, regional food scarcity will persist continually, particularly in South Asia, sub-Saharan Africa, the Middle East, and where population increase is rapid but agricultural outputs are low (FAO, 2017). Therefore, breeding and growing salt-tolerant crops to utilize the marginal and high-salinity soils are one of the most important strategies to meet the increase in food demand required by the estimated population in 2050 of 10 billion people (Mekonnen and Hoekstra, 2016; FAO, 2017; Morton et al., 2019).

MicroRNA (miRNA) is a non-coding single-stranded small RNA with a length of 21–24 nucleotides, and it acts as gene regulators to control the transcript abundance of its target gene. In the wild, miRNA exists in diverse organisms, including plants, animals, and microorganisms, and it regulates growth, development, signal transduction, response to adversity, and other biological processes. It was firstly discovered in *Caenorhabditis* (Lee and Ambros, 2001) and was then detected in four laboratories at approximately the same time in Llave et al. (2002), Mette et al. (2002), Park et al. (2002), Reinhart et al. (2002). After that, more and more plant miRNAs have been identified and functionally characterized in various plant species. MiRNA family names are listed in the order of publication, and miRNAs with similar sequences (usually fewer than 3 nt in difference) and common functions are classified as members of the same miRNA family (Wang Q. et al., 2014). Both the intraspecific conservation and interspecific differences of miRNAs are environmentally adaptive and evolve with the change in environment (Zhang et al., 2018). However, the evolution of miRNAs is conservative because some key target genes of miRNAs are conservative (Gramzow and Theißen, 2019).

Various enzymes and functional proteins are involved in the plant’s miRNA biosynthesis and functions. The primary miRNA transcripts for plants are produced by RNA polymerase II from miRNA genes, and these then pair with complementary bases to form special hairpin structures (Budak and Akpinar, 2015). Then, the stem ring secondary structure is generated by the DICER-LIKE1 (Bielewicz et al., 2013). After the methylation catalyzed by HUA Enhance 1 at the 3′ end, the double strand was transferred to the cytoplasm with the help of the transport protein HST. In the cytoplasm, this double-stranded miRNA is decomposed into mature single-stranded miRNA and integrated into RNA-induced silencing complex (RISC) cells, where miRNA interacts with the complementary target mRNA and activates the catalytic RISC with the assistance of Argonaute 1 (AGO1) (Koroban et al., 2016). There are two modes for miRNA to regulate gene expression: RNA cleavage and translation inhibition. The first mode is that miRNAs guide the Argonaute component of RISC to cleave a single phosphodiester bond opposite to the 10th and 11th nucleotides of the miRNA within complementary RNA. Then, the RISC will be free by releasing the fragments, and it then subsequently recognizes and cleaves another transcript (Jones-Rhoades et al., 2006). Afterward, the cleavage fragments are released to make the RISC competent for other RNA recognization and cleavage (Jones-Rhoades et al., 2006). MiRNA-mediated translational repression requires the participation of P-body components, a microtubule-severing enzyme, AGO1, and AGO10 (Brodersen et al., 2008). In addition, miRNA possibly prevents translation by triggering the sequestration of miRNA target in P-bodies (Chen, 2009). In addition, each miRNA can control multiple target genes (Haas et al., 2012). For instance, miR156 promotes floral meristem identity transformation by targeting SPL3, SPL4, and SPL5 in *Arabidopsis thaliana* (Xu et al., 2016). A gene can also be regulated by multiple miRNAs. For example, miR31 and miR143 affect steroid hormone synthesis by targeting the FSHR receptor (Zhang et al., 2019).

MiRNAs can regulate plant growth, development, pathogens, and abiotic stress responses. MiR160, miR169, peu-miRn68, and 477b are involved in the hormone signaling crosstalk model of root growth and development in apple rootstock, *A*. *thaliana* and *Populus* (Sorin et al., 2014; Lian et al., 2018; Meng et al., 2020). Cs-miR414 and cs-miR828 are involved in tea bud dormancy (Jeyaraj et al., 2014). For pathogen stress regulations, miR397 plays a negative regulatory role in apple resistance to hepatitis B virus (Yu et al., 2020), miR396 affects the susceptibility to rice blast (Chandran et al., 2019), and miR528 increases the viral defense ability of *Oryza sativa* (Wu et al., 2017). In the aspect of abiotic stress regulations, miR399 and miR827 are important for the resistance to phosphorus deficiency (Hackenberg et al., 2013; Du et al., 2018). The lack of sulfur induces the expression of miR395 for the regulation of genes in the sulfur assimilation pathway (Kawashima et al., 2009). The expression of miR319 is crucial for the cold tolerance of rice (Yang et al., 2013). MiR399 regulates *Arabidopsis* flowering at different temperatures (Kim et al., 2011). Recently, the comparative antagonistic expression profile of miR169 indicates that the miR169 family is a general regulator of various abiotic stresses (Rao et al., 2020). In addition, the over-expression of miR156 changes the expression level of other miRNAs, thus increasing the contents of anthocyanins, flavonoids, and flavonols and decreasing the total lignin content, suggesting the essential role of miRNAs in nutritional processes (Wang et al., 2020).

Noticeably, it is demonstrated that miRNA plays important roles in plant salinity responses and adaptation through various miRNA-mediated biological processes, including signal transduction, membrane transport, protein biosynthesis and degradation, photosynthesis, and transcription. In the present review, we mainly discuss the recent research progress on salt-stress-related miRNA in plants and the future research direction about miRNA in the salinity stress research field to come up with a strategy to improve the agronomic traits of stress tolerance through the manipulation of miRNAs.

## Identification and Expression of Plant miRNAs Under Salt Stress

In recent years, with the rapid development of biotechnology, such as microarray and high-throughput deep sequencing, thousands of plant miRNAs were identified under salt stress. As shown in Table 1, different concentrations (80–600 mM) of NaCl and treatment time (3 h to 15 days) were applied for salt stress treatments for identifying salt-responsive miRNA (Table 1). MiRNAs were detected in leaf, root, stem, and flower separately or in the whole seedling (Table 1). Fu et al. identified 1,077 miRNAs in *Zea mays*, comprising the highest number of identified miRNAs in various crops among the reports (Fu et al., 2017). Moreover, 882, 876, 693, and 650 miRNAs were identified in *Mesembryanthemum crystallinum*, *Medicago truncatula*, *Vicia faba*, and *Ipomoea batatas*, respectively (Jian et al., 2016; Cao et al., 2018; Alzahrani et al., 2019; Yang et al., 2020). The numbers of identified miRNA vary from dozens to hundreds, which may be due to the plant species, tissue specificity, development stage, and salt stress treatment methods. However, the large-scale identification of miRNAs under salt stress is very necessary and essential, and it lays a solid foundation for the further illumination of the miRNA network.

**TABLE 1:** The identification of plant miRNAs under salt stress by deep-sequencing.

**Latin name of sample**	**Sampling location**	**Salt stress treatment concentration/time**	**Number of miRNAs**	**References**
*Arabidopsis thaliana*	Root, bud	150 mM NaCl/7 d	118	Pegler et al., 2019
*Brassica juncea*	Seedling	150 mM Nacl, 200 mM NaCl/3 h, 6 h, 12 h, 24 h	51	Bhardwaj et al., 2014
*Brassica oleracea*	Flower	80 mM NaCl/15 d	81	Tian et al., 2014
*Cicer arietinum*	Root	150 mM NaCl/12 h	181	Kohli et al., 2014
*Cicer arietinum*	Root	250 mM NaCl/2 h	284	Khandal et al., 2017
*Eutrema salsugineum* 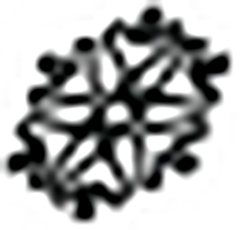	Seedlings	300 mM NaCl/0 h, 5 h, 12 h	99	Wu et al., 2016
*Glycine max*	Mature nodules	125 mM NaCl/6 h	238	Dong et al., 2013
*Halostachys caspica* 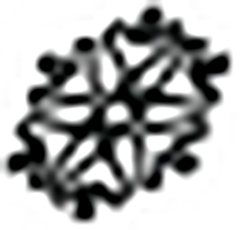	Root	600 mM NaCl/48 h	272	Yang et al., 2015
*Hordeum bulbosum*	Stem	250 mM NaCl/2 w	54	Liu and Sun, 2017
*Hordeum vulgare*	The plant body	100 mM NaCl/3 h, 8 h, 27 h	152	Deng et al., 2015
*Hordeum vulgare*	Seedling, leaves, roots	2% NaCl/-	259	Lv et al., 2012
*Ipomoea batatas*	Leaves, roots	150 mM NaCl/-	650	Yang et al., 2020
*Lagenaria siceraria(Molina)Standl*	Root	100 mM Nacl/4 h	91	Xie J. et al., 2015
*Leymus chinensis*	Seedling	100 mM NaCl and 200 mM NaHCO_3_/24 h	148	Zhai et al., 2014
*Linum usitatissimum*	-	50 mM NaCl/18 h	332	Yu et al., 2016
*Malvaceae Gossypium*	Leaves	150 mM Nacl/2 h, 4 h, 8 h	225	Yin et al., 2017
*Malvaceae Gossypium*	Seedling	0.5% NaCl/10 d	337	Xie F. et al., 2015
*Medicagosativa*	Root	300 mM NaCl/8 h	453	Long et al., 2015
*Medicago truncatula*	Seedling	20 mM NaCl + Na_2_SO4 5 mM Na_2_CO3 + NaHCO3/72 h	876	Cao et al., 2018
*Mesembryanthemum crystallinum* 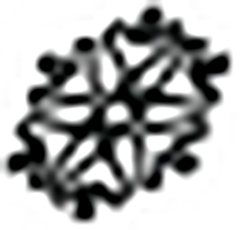	Seed	200 mM NaCl/60 h	967	Jian et al., 2016
*Mesembryanthemum crystallinum*	Seedling, root	200 mM NaCl/6 h	135	Chiang et al., 2016
*Musa nana*	Root	0mm (CTR), 100mm (TR100), and 300mm (TR300) NaCl/48 h	181	Lee et al., 2015
*Oryza glaberrima*	Leaves	200 mM NaCl/48 h	498	Mondal et al., 2018
*Oryza coarctata* 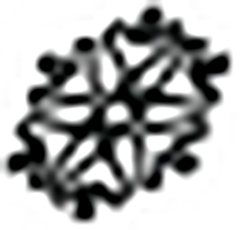	Root	450 mM NaCl/24 h	433	Mondal et al., 2015
*Oryza sativa*	Leaves	200 mM NaCl/15 d	357	Tripathi et al., 2018
*Oryza sativa*	Root, stem	256 mM NaCl/9 h	275	Parmar et al., 2020
*Panicumvirgatum*	Seedling	0.5% NaCl/10 d	273	Xie et al., 2014
*Paulownia*	Seedling	0.2%, 0.4% and 0.6% NaCl/20 d	187	Fan et al., 2016
*Phoenix dactylifera*	Seedling, leaves and roots	300 mM NaCl/72 h	422	Yaish et al., 2015
*Populus euphratica*	Leaves, roots	300 mM NaCl/3w	428	Si et al., 2014
*Populus tomentosa*	Seedling	200 mM NaCl/10 h	187	Ren et al., 2013
*Raphanus sativus*	Root	200 mM NaCl/3 h, 6 h, 12 h, 24 h, 48 h, 96 h	204	Sun et al., 2015
*Reaumuria soongorica* 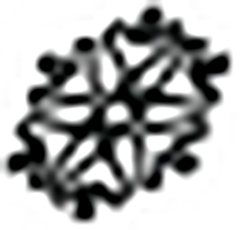	Seed	43, 273 mM NaCl/-	101	Zhang H. et al., 2020
*Rhizophora mangle, Heritiera littoralis*	Leaves	340 mM NaCl/96 h	147	Gharat and Shaw, 2015
*Saccharum officinarum*	Shoot, root	170 mM NaCl/-	131	Bottino et al., 2013
*Salicornia europaea*	Root, stem	200 mM NaCl/0 h, 12 h, 7 d	241	Feng et al., 2015
*Sesamum indicum*	Seedling	−/12 h, 24 h	442	Zhang Y. et al., 2020
*Solanum melongena*	Root	150 mM NaCl/24 h	98	Zhuang et al., 2014
*Spartina alterniflora* 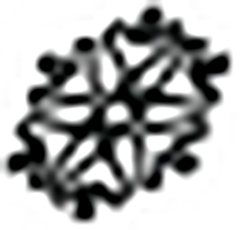	Leaf and root	500 mM sea salt/6, 12, 24, 72 h	902	Zandkarimi et al., 2015
*Suaeda maritima* 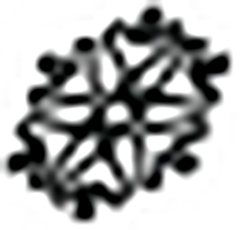	Aerial portions	255 mM NaCl/9 h	147	Gharat and Shaw, 2015
*Thellungiella salsuginea* 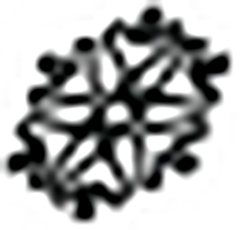	Leaves, roots	200 mM NaCl/24 h	246	Zhang et al., 2013
*Triticum aestivum*	Seedling	200 mM NaCl/7 d	317	Han et al., 2018
*Triticum monococcum subsp. monococcum*	Leaves, roots	100 mM NaCl/0, 3 h, 6 h, 12 h, 24 h	167	Ünlü et al., 2018
*Triticum turgidum ssp. dicoccoides*	The plant body	150 mM NaCl/0 h, 3 h, 6 h, 12 h, 24 h	212	Feng et al., 2017
*Vicia faba*	Seedling	150 mM NaCl/2 w	693	Alzahrani et al., 2019
*Zea mays*	Leaves and roots	250 mM NaCl/12 h	1077	Fu et al., 2017
*Zea mays*	Maize ears	**−/−**	102	Liu et al., 2014

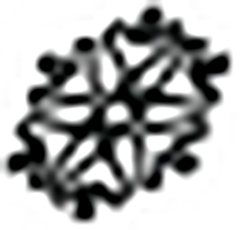
*indicates the plant name of halophyte; - indicates no related information.*

The expression levels of miRNA are up- or down-regulated by salinity stress. For instance, the expression of miR167 in panicle is negatively correlated with the increase of salt concentration (Jodder et al., 2018). In cotton, miR156, miR157, and miR172 are up-regulated at 0.25% NaCl, but their expression decreases with increasing salt concentration (Wang et al., 2013). The expression of miR164 also decreases with the increase of salt stress in maize (Shan et al., 2020). Macovei et al. found that the expression levels of Osa-miR414, -miR164e, and -miR408 significantly decrease with increased salt stress and further regulate the occurrence of genes to resist external salt stress by increasing the content of helicases (Macovei and Tuteja, 2012). In addition, some miRNAs are expressed differently in the early and late stages of salt stress treatment. For example, zma-miR169 displays initial up-regulation and subsequent down-regulation under salt stress (Luan et al., 2015). MiRNAs and their targets, such as cotton miR156-SPL2, miR159-TCP3, miR162-DCL1, miR395-APS1, and miR396-GRF1, exhibit negative correlation on expression levels (Wang et al., 2013).

Table 2 shows the expression levels of some representative miRNAs in plants under salt stress. MiR156, miR319, and miR528 are induced by salinity stress (Wang et al., 2013; Stief et al., 2014; Zhou and Luo, 2014; Xie F. et al., 2015; Yuan et al., 2015), while miR164 and miR397 are repressed (Macovei and Tuteja, 2012; Wang et al., 2013; Gupta et al., 2014; Qin et al., 2015; Xie F. et al., 2015; Lu et al., 2017), which were confirmed at least in two plant species (Table 2). Interestingly, the expression levels of nine miRNAs (e.g., miR159, miR168, miR169, miR172, miR393, miR395, miR396, miR399, and miR408) were promoted in some plant species but were inhibited in the other plant species. For instance, salinity stress increases the expression of miR393 in *Arabidopsis thaliana*, *Triticum aestivum*, and *Agrostis stolonifera*, but decreases the expression of miR393 *in Oryza sativa*, *Gossypium* sp., and *Spartina alterniflora* (Xia et al., 2012; Gupta et al., 2014; Iglesias et al., 2014; Qin et al., 2015; Xie F. et al., 2015; Zhao et al., 2019). Similarly, the expression of miR396 is increased by salinity in *Solanum lycopersicum*, *Nicotiana tabacum*, and *Agrostis stolonifera* but decreased in *Arabidopsis thaliana*, *Oryza sativa*, and *Spartina alterniflora* (Gao et al., 2010; Chen L. et al., 2015; Qin et al., 2015; Cao et al., 2016; Yuan et al., 2019). Up- or down-regulated gene expression usually suggests potential positive or negative functional role. However, the same miRNA has an opposite expression pattern in different plant species under salinity stress conditions, suggesting the same miRNA may play a diverse role in different plant species under salt stress. Moreover, the expression levels of some miRNAs, including miR167, miR390, miR394, miR402, and miR414 were only investigated in very few plant species under salinity stress (Table 2). Considering some miRNAs displayed totally different expressions in different species, their expression patterns need to be investigated in more plant species under salinity stress conditions.

**TABLE 2:** The expression of representative plant miRNAs under salt stress.

**MiRNA**	**Expression level**								
	*Arabidopsis thaliana*	*Oryza sativa*	*Solanum lycopersicum*	*Gossypium hirsutum*	*Zea mays*	*Triticum aestivum*	*Nicotiana tabacum*	*Agrostis stolonifera*	*Spartina alterniflora*
MiR156	↑Stief et al., 2014			Leaf (0-0.25%)↓, (0.25-0.5%)↑; Root (0-0.1%) ↑, (0.2-0.25%)↓, (0.25-0.5%)↑Wang et al., 2013	↑Kang et al., 2020		↑Kang et al., 2020		
MiR159				↑Xie F. et al., 2015; Wang et al., 2013		↓Wang B. et al., 2014			
MiR164	↓Lu et al., 2017	↓Macovei and Tuteja, 2012		↓Xie F. et al., 2015	↓Fu et al., 2017	↓Gupta et al., 2014			↓Qin et al., 2015
MiR167			↓Jodder et al., 2018	Leaf (0-0.1%)↑; Root (0.1-0.5%)↓, (0-0.1%)↑, (0.1-0.5%)↓Wang et al., 2013					
MiR168	↑Ding et al., 2009					↓Gupta et al., 2014			↓Qin et al., 2015
MiR169	↑Zhao et al., 2009			↓Yin et al., 2012	↓ (1-48h), ↑ (15d) Luan et al., 2014				↓Qin et al., 2015
MiR172				Leaf ↓; Root (0-0.1%)↓, (0.1-0.25%)↑, (0.25-0.5%)↓Wang et al., 2013		↑Gupta et al., 2014			
MiR319				↑Xie F. et al., 2015				↑Zhou and Luo, 2014	
MiR390				↓Yin et al., 2017					
MiR393	↑Iglesias et al., 2014	↓Xia et al., 2012		↓Xie F. et al., 2015		↑Gupta et al., 2014		↑Zhao et al., 2019	↓Qin et al., 2015
MiR394a	↑Song et al., 2013								
MiR394b	↑Song et al., 2013								
MiR395				Leaf (0-0.1%)↑; Root (0.1-0.5%)↓, (0-0.1%)↑, (0.1-0.5%)↓, Wang et al., 2013			↑Frazier et al., 2011		↓Qin et al., 2015
MiR396	↓Gao et al., 2010	↓Yuan et al., 2019	↑Cao et al., 2016	↑Wang et al., 2013			↑Chen L. et al., 2015	↑Yuan et al., 2019	↓Qin et al., 2015
MiR397				Leaf (0-0.25%) ↓, (0.25-0.5%) ↑Wang et al., 2013		↓Gupta et al., 2014			
MiR398	↓Jagadeeswaran et al., 2009			Leaf (0-0.25%)↓, (0.25-0.5%)↑; Root (0-0.1%)↑, (0.1-0.5%) ↓Wang et al., 2013		↓Wang B. et al., 2014	↑Leng et al., 2017		
MiR399	↑Guddeti et al., 2005			↓Wang et al., 2013					↓Qin et al., 2015
MiR402	↑Kim et al., 2010a								
MiR408	↑Guo et al., 2018	↓Macovei and Tuteja, 2012		↓Xie F. et al., 2015			↑Guo et al., 2018		
MiR414		↓Macovei and Tuteja, 2012							
MiR528		↑Yuan et al., 2015						↑Yuan et al., 2015	

*↑ and ↓ indicate the expressions of miRNAs are increased and decreased, respectively.% indicates the salt concentration.*

## miRNA Studies in Halophyte Palnts

Glycophyte plants, such as *Arabidopsis* and rice, can only survive at salinity levels 0–100 mM NaCl without any capability to adapt to high salt stress (Horie et al., 2012), whereas some remarkable halophytes can tolerate salinity levels as high as >1000 mM NaCl (Flowers and Colmer, 2008; Munns and Tester, 2008). To an extent, the salt-sensitive glycophytes may not provide enough insights into salt tolerance mechanisms, and the halophytes may have more value for expanding our knowledge about salt resistance mechanisms. Therefore, the exploration of the role of halophyte miRNAs in salinity adaptation can offer compelling contributions for devising strategies of resistance improvement in crops through genetic engineering and plant selection programs. However, there are not many reports on the discovery of salt-responsive miRNAs in halophytes (Table 1).

The halophyte plant *Suaeda maritima* grows naturally along the seashore. The expression of *S. maritima* sma-miR2 and sma-miR5 increases under the influence of seawater, suggesting their metabolic regulatory roles specific to saline environments (Gharat and Shaw, 2015). *Eutrema salsugineum*, a close relative of *A. thaliana*, can thrive in high salt conditions ranging from 100 to 500 mM (Amtmann, 2009). *E. salsugineum* has been developed as a valuable model plant for salt stress-tolerance study because its salinity tolerance is extreme, its lifetime is short, its seed production is copious, and its transformation is easy (Zhu, 2000; Amtmann et al., 2005). Zhang et al. (2013) identified 246 miRNAs candidates in *E. salsugineum*. In addition, 26 conserved miRNAs and 4 novel miRNAs were found to display a significant response to salt stress in *E. salsugineum* (Zhang et al., 2013; Wu et al., 2016). Recently, 88 conserved miRNAs and 13 novel miRNAs were identified from *Reaumuria soongorica* seeds treated with various NaCl concentrations, providing a useful reference for salt resistance improvement of seed germination (Zhang H. et al., 2020). A total of 135 conserved miRNAs and the hairpin precursor of 12 novel mcr-miRNAs were found from *M. crystallinum* seedlings treated with 200 mM NaCl (Chiang et al., 2016). *Oryza coarctata* is a wild relative of rice and grown in saline water. Mondal et al. found 338 known and 95 novel miRNAs in salt-treated *O. coarctata* leaves, providing a miRNA-target networking that is involved in salt stress adaption (Mondal et al., 2015). *Halostachys caspica* (Bieb.), a salt-tolerant short shrub, can be naturally grown on the field with a salt concentration as high as 100 g/kg dry soil (Song et al., 2006). (Yang et al., 2015) found that 31 conserved miRNAs and 12 novel miRNAs were significantly up-regulated, and 48 conserved miRNAs and 13 novel miRNAs were significantly down-regulated by salinity stress in *H. caspica.* A set of miRNAs were also identified in a salt marsh monocot halophyte smooth cordgrass (*Spartina alterniflora* Loisel) and another plant named salt cress (*Thellungiella salsuginea*) (Zhang et al., 2013; Zandkarimi et al., 2015). These identified miRNAs in halophytes can be further projected as potential miRNAs for developing salt tolerance in glycophyte crops.

## Functions of miRNA Under Salt Stress

Numerous plant miRNAs have been identified under salt stress, but not many miRNAs have been functionally characterized in detail. Table 3 shows us the miRNAs responsive to salt stress, and these which were functionally studied by transgenetic approaches, such as overexpression and knocked down/out of the miRNA itself or its targets (Table 3). For instance, miR394a/b over-expression and *lcr* (functional loss of miR394 target LCR) mutant plants are hypersensitive to salt stress, but LCR over-expressing plants display the salt-tolerant phenotype (Song et al., 2013). MiR393 is a comparative well-studied plant miRNA in different plant species, including *Arabidopsis*, rice, and creeping bentgrass. MiR393ab mutant shows reduced inhibition of LR (lateral root) number and length, increased levels of ROS in LRs, and reduced APX enzymatic activity (Iglesias et al., 2014). Over-expressing Osa-mR393 in rice and *Arabidopsis* reduces tolerance to salt and drought and increases tillers and early flowering (Gao et al., 2011; Xia et al., 2012), while over-expressing miR393-resistant form mTIR1 in *Arabidopsis* enhances salt tolerance in mTIR1 transgenic plant (Chen Z. et al., 2015). However, over-expressing Osa-miR393a in creeping bentgrass improves salt stress tolerance associated with the increased uptake of potassium (Zhao et al., 2019), suggesting that the same miRNA or different miRNA from the same miRNA family may have different promotion and inhibition effects on salt tolerance in different plants. A similar situation was found for miRNA396, that is, over-expressing Osa-miR396c reduced salt and alkali stress tolerance in rice and *Arabidopsis* (Gao et al., 2010), but enhanced salt tolerance associated with improved water retention, increased chlorophyll content, cell membrane integrity, and Na^+^ exclusion during high salinity exposure in creeping bentgrass (Yuan et al., 2019). Additionally, over-expressing Sp-miR396a-5p in tobacco enhanced its tolerance to salt, drought, and cold stresses (Chen L. et al., 2015). The overexpression of miR395c or miR395e retarded and accelerated, respectively, the seed germination of *Arabidopsis* under high salt or dehydration stress conditions (Kim et al., 2010b).

**TABLE 3:** The functions of miRNA under salt stress.

**Species**	**Common Name**	**MiRNA name**	**Target gene**	**Salt tolerance phenotype**	**Method/Technology**	**References**
*Malus domestica*	Apple	MiR156a	*MdSPL13*	Overexpressing MiR156a weakened salt resistance in apple, whereas MdSPL13 strengthened	MiR156a and SPL13 overexpression	Ma et al., 2020
*Populus euphratica*		Peu-miR164	*PeNAC070, PeNAC012, PeNAC028*	Promoted lateral root development, delayed stem elongation, and increased sensitivity to drought and salt stresses in PeNAC070 transgenic plants	Overexpress PeNAC070 in *Arabidopsis*	Lu et al., 2017
*Glycine max*	Soybean	MiR169	*GmNFYA3*	Reduced leaf water loss, enhanced drought tolerance and increased sensitivity to high salinity and exogenous ABA in GmNFYA3 overexpression plants	Overexpress GmNFYA3 in *Arabidopsis*	Ni et al., 2013
*Glycine max*	Soybean	Gma-miR172c	*Glyma01g39520*	Soybean miR172c confers tolerance to water deficit and salt stress, but increases ABA sensitivity in transgenic *Arabidopsis thaliana*	Overexpress of soybean miR172c	Li et al., 2016
*Glycine max*	Soybean	MiR172c	*NNC1*	Overexpression and knockdown of miR172c activity resulted in substantially increased and reduced root sensitivity to salt stress, respectively	Overexpress miR172c and knockdown miR172c	Sahito et al., 2017
*Agrostis stolonifera*	Creeping bentgrass	Osa-miR319a	*AsPCF5, AsPCF6, AsPCF8, AsTCP14*	Enhanced drought, salt tolerance, increased leaf wax content and water retention, but reduced sodium uptake	Overexpressing Osa-miR319a in creeping bentgrass	Zhou and Luo, 2014; Zhou et al., 2013
*Panicum virgatum*	Switchgrass	Osa-miR319b	*PvPCF5*	Osa-miR319b positively regulated ET synthesis and salt tolerance	Overexpress Osa- miR319b, target mimic miR319 in swithgrass	Liu et al., 2019
*Populus* spp.	Poplar	MiR390	*ARF3.1, ARF3.2, ARF4*	Stimulated LR development and increased salt tolerance	Overexpress and knockdown (STTM) miR390 in poplar	He et al., 2018
*Helianthus tuberosus*	Jerusalem artichoke	MiR390	*TAS3, ARF3/4*	May play an active role in salt tolerance	Bioinformatics, gene cloning and RT-qPCR analyses	Wen et al., 2020
*Arabidopsis thaliana*	*Arabidopsis*	MiR393	*TIR1, AFB2*	MiR393ab mutant shows reduced inhibition of LR number and length, increased levels of ROS in LRs, and reduced APX enzymatic activity	miR393ab double mutant was obtained from the cross of miR393a-1 and miR393b-1	Iglesias et al., 2014
*Arabidopsis thaliana*	*Arabidopsis*	MiR393	*TIR1*	Enhanced salt tolerance in mTIR1 transgenic plant	Overexpressing miR393-resistant form mTIR1 in *Arabidopsis*	Chen Z. et al., 2015
*Oryza sativa*	Rice	OsmiR393	*OsTIR1, OsAFB2*	Reduced tolerance to salt and drought, increased tillers and early flowering	Overexpressing OsmiR393 in rice	Xia et al., 2012
*Oryza sativa*	Rice	Osa-miR393	*LOC_Os02g06260, LOC_Os05g41010, LOC_Os05g05800*	Transgenic plants were more sensitive to salt and alkali treatment	Overexpressing Osa-miR393 in rice and *Arabidopsis*	Gao et al., 2011
*Agrostis stolonifera*	Creeping bentgrass	Osa-miR393a	*AsTIR1, AsAFB2*	Improved salt stress tolerance associated with increased uptake of potassium	Overexpressing Osa-miR393a in creeping bentgrass	Zhao et al., 2019
*Arabidopsis thaliana*	*Arabidopsis*	MiR394a/b	*LCR*	MiR394a/b over-expression and *lcr* (LCR loss of function) mutant plants are hypersensitive to salt stress, but LCR over-expressing plants display the salt-tolerant phenotype	Overexpressing miR394a/b and LCR in *Arabidopsis*	Song et al., 2013
*Arabidopsis thaliana*	*Arabidopsis*	MiR395c, MiR395e	*APS1, APS3, APS4, SULTR2;1*	Overexpression of miR395c or miR395e retarded and accelerated, respectively, the seed germination of *Arabidopsis* under high salt or dehydration stress conditions	Overexpression of miR395c or miR395e in *Arabidopsis*	Kim et al., 2010b
*Oryza sativa*	Rice	Osa-miR396c	*LOC_Os01g32750, LOC_Os02g45570, LOC_Os04g5119*	Reduced salt and alkali stress tolerance	Overexpressing osa-miR396c in rice and *Arabidopsis*	Gao et al., 2010
*Agrostis stolonifera*	Creeping bentgrass	Osa-miR396c	*GRF*	Enhanced salt tolerance associated with improved water retention, increased chlorophyll content, cell membrane integrity, and Na^+^ exclusion during high salinity exposure	Overexpressing Osa-miR396c in creeping bentgrass	Yuan et al., 2019
*Solanum pimpinellifolium*	Tomato	Sp-miR396a-5p	*GRF1,GRF3, GRF7,GRF8*	Enhanced its tolerance to salt, drought and cold stresses	Overexpressiing Sp-miR396a-5p in tobacco	Chen L. et al., 2015
*Arabidopsis thaliana*	*Arabidopsis*	MiR399f	*ABF3, CSP41b*	Plants overexpressing miR399f exhibited enhanced tolerance to salt stress, but hypersensitivity to drought	Overexpressing miR399f in *Arabidopsis*	Baek et al., 2016
*Arabidopsis thaliana*	*Arabidopsis*	MiR402	*DEMETER-LIKE protein3*	Accelerated the seed germination and seedling growth of *Arabidopsis* under salt stress conditions	Overexpression of miR402 in *Arabidopsis*	Kim et al., 2010a
*Arabidopsis thaliana*	*Arabidopsis*	MiR408	*Plantacynin, Cupredoxin, Uclacyanin, LAC3*	Improved tolerance to salinity, cold and oxidative stress, but enhanced sensitivity to drought and osmotic stress	Overexpressing miR408 in *Arabidopsis*	Ma et al., 2015
*Triticum aestivum*	Wheat	Tae-miR408	*TaCLP1*	Significantly increased cell growth under high salinity and Cu^2+^ stresses	Overexpressing TaCLP1 in yeast	Feng et al., 2013
*Triticum aestivum*	Wheat	TaemiR408	*TaCP,TaMP,TaBCP, TaFP,TaKRP,TaABP*	Enhanced stress tolerance, improved phenotype, biomass, and photosynthesis behavior under salt treatments	Overexpressing TaemiR408 in tobacco	Bai et al., 2018
*Salvia miltiorrhiza*	-	Sm-miR408	*Copper-binding proteins, Laccase*	Promoted seed germination and reduced the accumulation of ROS under salt stress, positive responses to salt tolerance	Overexpressing Sm-miR408 in tobacco	Guo et al., 2018
*Gossypium* spp.	Cotton	MiR414c	*GhFSD1*	Overexpressing miR414c increased sensitivity to salinity stress, yielding a phenotype similar to that of GhFSD1-silenced cotton	Silence *GhFSD1* in cotton, overexpressing ghr- miR414c and *GhFSD1* in *Arabidopsis*	Wang et al., 2019
*Arabidopsis thaliana*	*Arabidopsis*	MiR417	*At1g04150, At1g17730, At5g66460, At5g49680, At4g11130, At1g48310, At3g06400, At1g19850*	Seed germination of the transgenic plants was retarded under high salt condition	Overexpress miRNA417 in *Arabidopsis*	Jung and Kang, 2007
*Agrostis stolonifera*	Creeping bentgrass	Osa-miR528	*AsAAO, AsCBP1*	Shortened internodes, increased tiller number, and upright growth, enhances tolerance to salinity stress and nitrogen starvation	Overexpressing Osa-miR528 in creeping bentgrass	Yuan et al., 2015
*Gossypium hirsutum*	Cotton	MiRNVL5	*GhCHR*	*Arabidopsis* constitutively expressing miRNVL5 showed hypersensitivity to salt stress	Ectopic expression of miRNVL5 and GhCHR in *Arabidopsis*	Gao et al., 2016

Over-expressing miR156a weakens salt resistance in apples, whereas its target gene MdSPL13 strengthens salt resistance (Ma et al., 2020). Transgenic *Arabidopsis* plants over-expressing the target gene PeNAC070 of miR164 exhibits promoted LR development, delayed stem elongation, and increased sensitivity to salt stress (Lu et al., 2017). Over-expressing the target gene GmNFYA3 of miR169 reduces leaf water loss, enhances drought tolerance, and increases sensitivity to high salinity and exogenous ABA (Ni et al., 2013). Over-expression of miR172c substantially increased the sensitivity of plant roots to salt stress, and the removal of miR172c would decrease the sensitivity of plant roots to salt stress, respectively (Li et al., 2016; Sahito et al., 2017). Osa-miR319a and mi319b positively regulate salt tolerance in creeping bentgrass and swithgrass, respectively (Zhou et al., 2013; Zhou and Luo, 2014; Liu et al., 2019). MiR390 increases LR growth under salt stress via the auxin pathway (He et al., 2018). Additionally, over-expressing miR399f, miR402, and miR408 in *Arabidopsis*, Tae-miR408 and Sm-MIR408 in tobacco, and Osa-miR528 in creeping bentgrass increases salinity tolerance (Kim et al., 2010a; Feng et al., 2013; Ma et al., 2015; Yuan et al., 2015; Baek et al., 2016; Bai et al., 2018; Guo et al., 2018), indicating that these miRNAs enhance plant salt stress adaptation. By contrast, over-expressing miR414c, miR417, and miRNVL5 increases sensitivity to salinity stress (Jung and Kang, 2007; Gao et al., 2016; Wang et al., 2019). Collectively, these results suggest that the agronomic trait of salinity stress tolerance could be enhanced by the manipulation of miRNA or its target.

## Discussion and Future Prospects

In the face of soil salinization, the cultivation of saline-tolerant plants is one of the most economical and effective technologies for biological improvement. Understanding the molecular mechanisms of miRNAs in abiotic stress provides an effective tool for plant breeding, especially in the context of climate and human-induced environmental changes. The essential regulating role of miRNAs in plant salt stress response reveals that miRNA could be applied for salt resistance improvement in crops. The salinity resistance of transgenic plants can be remarkably increased by over-expressing miRNA or knocking down/out the target gene of miRNA. Alternatively, the salinity resistance can be promoted by knocking down/out miRNA, which has a negative effect on salinity response, or over-expressing the target gene of the miRNA. Considering that one miRNA may have more than one targets that would cause totally different effects on plants, we should carefully consider the miRNA effects on crop growth, development, and the sensitivity to other abiotic stresses when optimizing the salinity resistance by miRNA manipulation.

The homologous tetraploid was more tolerant to salt stress than the diploid. Moreover, novel miRNAs induced by genome replication were identified, suggesting salt-responsive miRNAs could be screened by comparative analysis on the plant materials with different ploidy and salinity stress tolerance to explain the key roles of miRNA in achieving better salt stress tolerance. Generally, miRNAs are evolutionarily conserved in their functions in response to salt stress. However, the same miRNAs or different miRNAs from the same miRNA family may have different promotion and inhibition effects on salt tolerance in different plants. Therefore, the function of some miRNAs should be widely studied in different species, especially in crops.

Moreover, considering the significant number of salt- stress-responsive miRNAs identified by using powerful technology (such as high throughput sequencing), only a few miRNAs have been functionally characterized. Therefore, after the identification of plant miRNAs under salinity stress, further studies should be focused on the exploration of function, which will be very crucial for the salt tolerance improvement through miRNA manipulation in crops. Additionally, miRNAs may affect the plant stress tolerance through their interaction with ABA biosynthesis and the regulation of auxin response factors, The investigation of the crosstalk between miRNA and plant hormone will thus expand our knowledge and understanding of the role of plant miRNAs under stress conditions. Finally, the construction of the plant miRNA network in salt stress response will shed light on the salinity resistance improvement through miRNA manipulation in crops.

## Author Contributions

TX conceived and designed this manuscript. TX, LZ, and ZY wrote the manuscript. YW and TD helped to revise the manuscript. All authors read and approved the manuscript.

## Conflict of Interest

The authors declare that the research was conducted in the absence of any commercial or financial relationships that could be construed as a potential conflict of interest.
